# Insights into N6-methyladenosine (m6A) modification of noncoding RNA in tumor microenvironment

**DOI:** 10.18632/aging.204679

**Published:** 2023-05-12

**Authors:** YanJun Zhang, Lijuan Zhan, Jing Li, Xue Jiang, Li Yin

**Affiliations:** 1 College of Pharmacy and Traditional Chinese Medicine, Jiangsu College of Nursing, Huaian, Jiangsu 223005, China; 2 Department of Biopharmaceutics, Yulin Normal University, Guangxi, Yulin 537000, China; 3 Bioengineering and Technology Center for Native Medicinal Resources Development, Yulin Normal University, Yulin 537000, China

**Keywords:** m6A, ncRNA, tumor microenvironment, tumor metastasis, exosome, biomarker, targeted therapy

## Abstract

N6-methyladenosine (m6A) is the most abundant RNA modification in eukaryotes, and it participates in the regulation of pathophysiological processes in various diseases, including malignant tumors, by regulating the expression and function of both coding and non-coding RNAs (ncRNAs). More and more studies demonstrated that m6A modification regulates the production, stability, and degradation of ncRNAs and that ncRNAs also regulate the expression of m6A-related proteins. Tumor microenvironment (TME) refers to the internal and external environment of tumor cells, which is composed of numerous tumor stromal cells, immune cells, immune factors, and inflammatory factors that are closely related to tumors occurrence and development. Recent studies have suggested that crosstalk between m6A modifications and ncRNAs plays an important role in the biological regulation of TME. In this review, we summarized and analyzed the effects of m6A modification-associated ncRNAs on TME from various perspectives, including tumor proliferation, angiogenesis, invasion and metastasis, and immune escape. Herein, we showed that m6A-related ncRNAs can not only be expected to become detection markers of tumor tissue samples, but can also be wrapped into exosomes and secreted into body fluids, thus exhibiting potential as markers for liquid biopsy. This review provides a deeper understanding of the relationship between m6A-related ncRNAs and TME, which is of great significance to the development of a new strategy for precise tumor therapy.

## INTRODUCTION

The tumor microenvironment (TME) refers to the environment in which tumor cells grow, that includes the tumor cells, surrounding fibroblasts, immune and inflammatory cells, stromal cells, and other cells, as well as the extracellular matrix (ECM), microvessels and biomolecules in the nearby area [1, 2]. In contrast to the normal microenvironment, TME is characterized by low oxygen content, acid accumulation, and abnormal local immune status, which are conducive for proliferation, invasion, adhesion, angiogenesis, resistance to radiotherapy and chemotherapy, and emergence of malignant tumors. TME is a dynamic regulatory network involving multiple signals, in which a variety of cellular and molecular pathways can be potential therapeutic targets. Important anti-tumor therapeutic strategies involve blocking tumor-associated pathways in TME, inhibiting the activity of cells with tumor-enhancing effects, and promoting anti-tumor immunity, in combination with traditional treatments, such as surgery, chemotherapy, and radiotherapy. The molecular substances in TME are closely related to the pathogenesis and development of tumor and are potentially sensitive and specific tumor markers.

There are more than 100 chemical modifications of RNA, with methylation being the main form of modification in all types of RNA [3]. RNA methylation accounts for >60% of all RNA modifications. N6-methyladenosine (m6A) is the most abundant RNA modification in eukaryotes, and regulates the post-transcriptional expression of genes. m6A is mainly distributed in the protein coding sequence of mRNA, 3′ untranslated region (UTR), region around the stop codon, and long exon region [4, 5]. The role of m6A modification in the regulation of gene expression is closely related to various normal physiological processes, including cell differentiation, DNA damage response, biological clock, and sex determination, and the occurrence and development of diseases, such as tumors [5]. During tumor development, m6A modification can regulate the expression of oncogenes and tumor suppressor genes in tumor cells, thereby regulating tumor angiogenesis, extracellular matrix remodeling, epithelial-mesenchymal transition (EMT), and the immune microenvironment to promote tumorigenesis [6]. Recent research has found that m6A modification regulates the transcription level of mRNA encoding genes and also affects the transcription and generation of a variety of non-coding RNAs (ncRNAs) (such as microRNAs, lncRNAs, and circRNAs) [3]. Therefore, m6A modification participates in the regulation of various life processes in cells at multiple levels and plays a regulatory role in many diseases, including tumors [6].

This review aimed to summarize the biological characteristics of ncRNAs related to m6A modification and their roles in TME formation, explore the application prospects of ncRNAs related to m6A modification in clinical treatment, and provide new strategies for the accurate diagnosis and treatment of tumors.

## Tumor microenvironment

**Figure 1:**
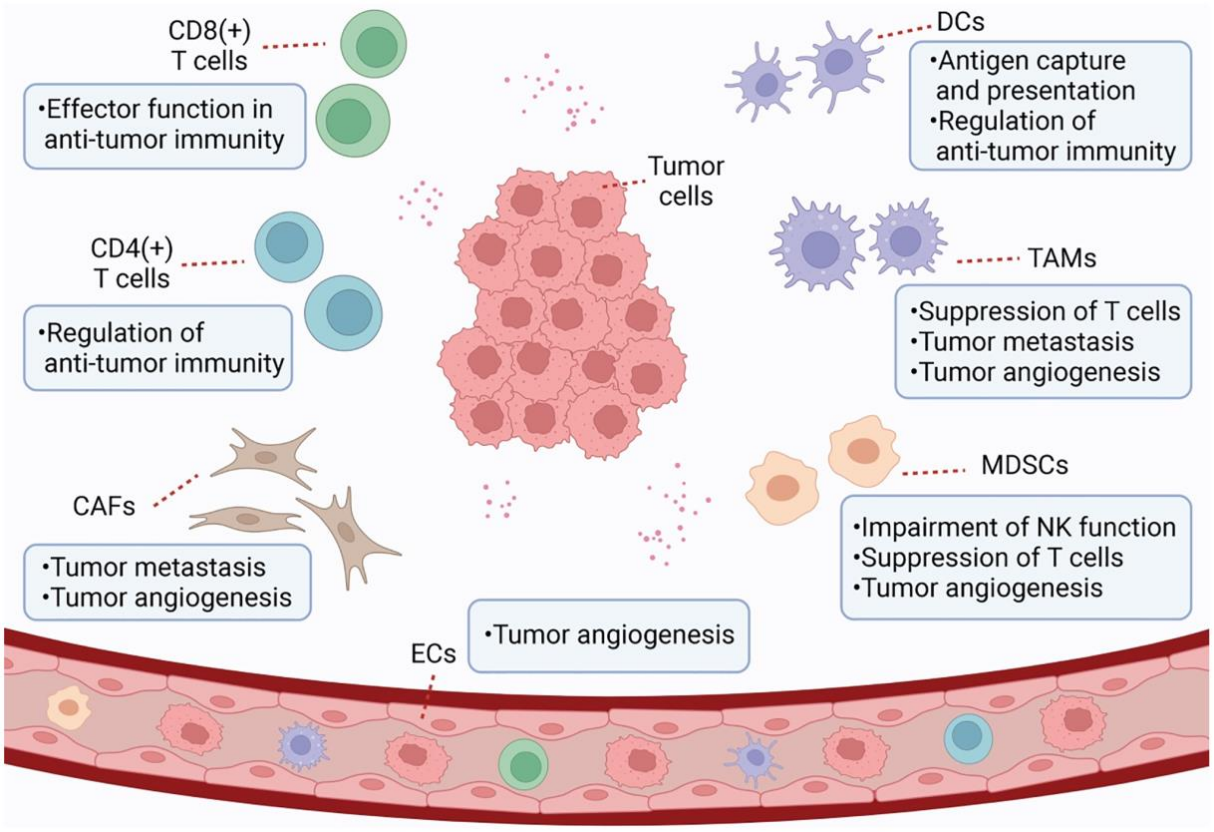
**Schematic representation of TME.** TME is a complex and comprehensive system. In addition to tumor cells, stromal cells (e.g., CAF and tumor-associated endothelial cells) and immune cells (e.g., MDSC, TAM, DC, and TILs) are also important components of TME.

Since Stephen Paget proposed the hypothesis of “seed and soil” theory of tumors in 1889, the relationship between tumors and TME has attracted extensive research attention. During the occurrence and development of tumor, TME can interact with tumor cells and play a key role in tumor proliferation, inflammation, immune evasion, metastasis and drug resistance. TME is a complex integrated system, and tumor and stromal cells (such as cancer-associated fibroblasts and tumor-related endothelial cells) and immune cells (such as myeloid-derived suppressor cells, tumor-associated macrophages, dendritic cells, and tumor-infiltrating lymphocytes) in TME can communicate in several ways to promote tumor growth, angiogenesis, immune escape, and metastasis (Figure 1). As an important epigenetic regulatory mechanism, ncRNAs can regulate gene expression at the genomic and chromosomal levels [7, 8], participate widely in signaling, and be transmitted through exosomes to influence recipient cells and promote TME formation.

A study by Tikhonova [3] profiled 17,374 single cells of the mouse bone marrow niche, identified previously unrecognized heterogeneity within the bone marrow microenvironment and demonstrate how the microenvironment responds to acute bone marrow stress at a single-cell level. We couple transcriptional profiling with fluorescent reporters and functional studies to demonstrate that vascular expression of the Notch receptor DLL4—identified by our single-cell studies—suppresses premature upregulation of the myeloid program in HSCs. They showed that single-cell transcriptomic profiling can translate to mechanistic insights into the regulation of the differentiation of stem and progenitor cells. They match haematopoietic factors to their cellular sources, and show that the loss of the vascular-endothelial-expressed Notch ligand DLL4 skews bone marrow haematopoiesis towards a significant transcriptional reprogramming and myeloid priming of HSPCs. Future studies are needed to investigate the functional consequences of niche heterogeneity on aberrant stem cell functions, such as immunodeficiency, haematopoietic malignancies and ageing. Targeting niche-associated factors could interfere with the initiation and progression of disease, as has recently been shown by targeting vascular CXCR4–CXCL12 interactions in acute lymphoblastic leukaemia [5].

A study by Zhou [6] conducted single cell RNA sequencing of human and mouse HCC tumors revealed heterogeneity of cancer-associated fibroblast (CAF). Cross-species analysis determined the prominent CD36+ CAFs exhibited high-level lipid metabolism and expression of macrophage migration inhibitory factor (MIF). Lineage-tracing assays showed CD36+CAFs were derived from hepatic stellate cells. Furthermore, CD36 mediated oxidized LDL uptake-dependent MIF expression via lipid peroxidation/p38/CEBPs axis in CD36+ CAFs, which recruited CD33+myeloid-derived suppressor cells (MDSCs) in MIF- and CD74-dependent manner. Co-implantation of CD36+ CAFs with HCC cells promotes HCC progression *in vivo*. Finally, CD36 inhibitor synergizes with anti-PD-1 immunotherapy by restoring antitumor T-cell responses in HCC. The work underscores the importance of elucidating the function of specific CAF subset in understanding the interplay between the tumor microenvironment and immune system.

### Stromal cells in tumor microenvironment

Stromal cells in TME can be induced by signal factors secreted by tumor cells, and their gene expression and functional status are different from those of stromal cells in normal tissue. Stromal cells promote tumor progression by participating in the proliferation and migration of tumor cells, extracellular matrix remodeling, immune cell recruitment, and tumor angiogenesis.

There are numerous cancer-associated fibroblasts (CAFs) in TME. CAF can be derived from local normal fibroblasts, differentiated from bone marrow-derived mesenchymal cells, or generated by transdifferentiation of pericytes and other cell types [9, 10]. Transforming growth factor β (TGF-β), platelet-derived growth factors (PDGFs), and fibroblast growth factor (FGF) 2 are the main inducers of CAF activation [11]. Stromal derived factor 1 (SDF-1) secreted by CAF binds to CXCR4 on the surface of tumor cells and vascular endothelial cells (ECs), increasing tumor growth and malignancy as well as promoting angiogenesis [12]. Hepatocyte growth factor secreted by CAF enhances tumor invasion and metastasis by activating c-Met [13]. CAF can also induce the transcription of long non-coding RNA (lncRNA) HOTAIR by secreting TGF-β1, thereby promoting EMT and the metastasis of breast cancer cells [14]. In ovarian cancer, CAF upregulates lncRNA LINC00092 by secreting CXCL14 and promotes cancer metastasis by altering glycolysis [15].

Angiogenesis plays an important role in tumor growth and metastasis. Mesenchymal stem cells, tumor-associated macrophages and CAF secrete a variety of vascular growth factors into TME to promote angiogenesis and EC proliferation is the core process for neovascularization. CXCR7 expression is upregulated in tumor-associated EC, which promotes angiogenesis in TME through ERK1/2 phosphorylation [16]. Exosomes containing miR-23a secreted by tumor cells promote tumor microvasculogenesis by acting on SIRT1 in EC [17]. The vascular endothelial growth factor (VEGF)/VEGFR signaling pathway is important in tumor angiogenesis. VEGF upregulates Bcl-2 in vascular ECs and promotes tumor angiogenesis [18]. miR-134 inhibits angiogenesis in osteosarcoma by targeting the VEGF/VEGFR1 pathway [19]. In neuroblastoma, MALAT1 promotes EC migration and angiogenesis through the upregulation of FGF2 [20]. In addition, tumor cells and ECs directly interact to promote tumor angiogenesis through the mitogen-activated protein kinase (MAPK) and Notch pathways [21, 22]. Currently, anti-angiogenesis therapy is a conventional treatment strategy for tumor in clinical practice.

### Immune cells in tumor microenvironment

During tumor development, tumor cells can take advantage of the negative regulation mechanism of the immune system to alter the functional status of various infiltrated immune cells, forming a microenvironment with low anti-tumor immunity, leading to tumor immune tolerance and immune escape. Therefore, inducing and enhancing the anti-tumor immune response is an important strategy to improve anti-tumor therapy efficacy.

TME comprises numerous immune cells, including myeloid-derived suppressor cells (MDSCs) that are a group of heterogeneous cells that originate from the myeloid system. MDSCs continue to differentiate into dendritic cells, macrophages, and granulocytes under normal circumstances, while under pathological conditions, MDSCs cannot differentiate but they develop into a cell population that can inhibit immune function. Several cytokines in TME can induce the proliferation of MDSCs, including COX2, interleukin6 (IL-6), granulocyte macrophage colony stimulating factor (GM-CSF), and VEGF [23]. Lnc-CHOP enhances the immunosuppressive function of MDSCs by promoting C/EBPβ activation and the expression of molecules associated with MDSC immunosuppressive activity [24]. However, lnc-C/EBPβ inhibits the activation of C/EBPβ and reduces MDSC function [25]. The immunosuppressive function of MDSCs mainly manifests as the suppression of the immune-killing effect of T cells and NK cells. MDSCs can inhibit T cells by promoting T cell apoptosis, consuming essential amino acids for T cell function, reducing T cell migration to lymph nodes, preventing T cell signaling, and inducing T cell differentiation imbalance [26, 27]. MDSCs inhibit NK cells by inhibiting activation receptor expression, downregulating perforin secretion, and limiting response to IL-2 by NK cells [28]. MDSCs are also involved in the regulation of tumor angiogenesis [29].

Macrophages are classified into M1 and M2 phenotypes [30]. M1 macrophages can secrete pro-inflammatory factors, such as IL-1, IL-12, and TNF-α, which are involved in defense against infections and tumoricidal activities [31]. In contrast, M2 macrophages highly express the immunosuppressive factor IL-10, which exerts anti-inflammatory and tumor-promoting effects [32]. Tumor-associated macrophages (TAMs) infiltrate the tumor area, showing a functional phenotype similar to that of M2 macrophages. A variety of chemokines, growth factors, and cytokines in TME recruit macrophages to infiltrate [33] and induce M2 phenotype transformation. TAM recruitment and polarization are regulated by ncRNAs. lncRNAs LNMAT1 [34] and lnc-BM [35], promote macrophage recruitment, while lncRNA-MM2P regulates the expression of M2-related genes in macrophages [36]. miR-21-3p, miR-125b-5p and miR-181d-5p can be delivered into TME by cancer-derived exosomes to promote M2 polarization of macrophages [37]. In TME, TAM can secrete matrix metalloproteinases (MMP) to promote basement membrane degradation, thus promoting metastasis. TAM can also secrete basic FGF, VEGF, CXCL8, and other factors that promote tumor angiogenesis [38]. TGF-β and IL-10 released by M2 macrophages can inhibit T cell immune responses and anti-tumor immunity.

Dendritic cells (DCs) have a strong ability for antigen uptake and processing, which can induce an anti-tumor immune response by presenting tumor antigens. DCs are divided into plasmacytoid dendritic cells (pDCs) and myeloid dendritic cells (mDCs). pDCs exert non-specific anti-infection and anti-tumor immunity by activating monocytes, macrophages, B cells, NK cells, and naive T cells [37]. mDCs mainly activate the transformation of initial CD4 cells to T helper (Th) 1 cells and CD8 cells to cytotoxic T lymphocytes (CTL) through antigen presentation and exert specific anti-infection and anti-tumor immunity [39]. Lnc-DC can bind to STAT3 to promote DC differentiation and improve the antigen presentation capacity [40]. However, in TME, various cytokines can induce DC differentiation disorders, resulting in abnormal immunomodulatory functions.

In TME, tumor-infiltrating lymphocytes (TILs) are influenced by different cytokines and activation mechanisms to produce different immune responses. CD8+T cells can recognize tumor antigens or secrete cytokines and play an effector-killing role in anti-tumor immunity. Lnc-Tim3 interacts with Tim-3 to release Bat3, resulting in CD8+ T-cell depletion and immune evasion in hepatocellular carcinoma (HCC) [41]. Activated CD8+T cells express cytotoxic T lymphocyte-associated antigen-4 (CTLA-4) and programmed death-1 (PD-1) [42]. Immunocheckpoint inhibitors (ICIs) targeting CTLA-4 and PD-1 have become important anti-tumor immunotherapies. The overexpression of miR-142-5p in tumor cells can block the PD-L1/PD-1 pathway and enhance anti-tumor immune function [43]. CD4+T cells assist and regulate immunity and can proliferate and differentiate into a variety of cell subsets: Th1, Th2, Th17, and regulatory T cells (Tregs) [44]. Interferon (IFN)-γ secreted by Th1 cells can activate cytotoxic CD8+T cells, DCs, and macrophages and play an anti-tumor role. Th2 cells secrete IL-4 to activate the tumor-promoting macrophages; moreover, Tregs can inhibit the proliferation and differentiation of T cells, hinder antigen presentation, mediate target cell death, and play an immunosuppressive part [45]. Several chemokines in TME and DC differentiation disorder can promote the recruitment, proliferation and activation of Tregs [46, 47]. The lncRNA SNHG1 regulates Treg cell differentiation through the miR-448/IDO pathway, affecting tumor immune escape [48], while specific Treg clearance can enhance the anti-tumor immune response [49, 50].

## m6A modifications and noncoding RNAs

### Molecular component of m6A RNA methylation

**Figure 2:**
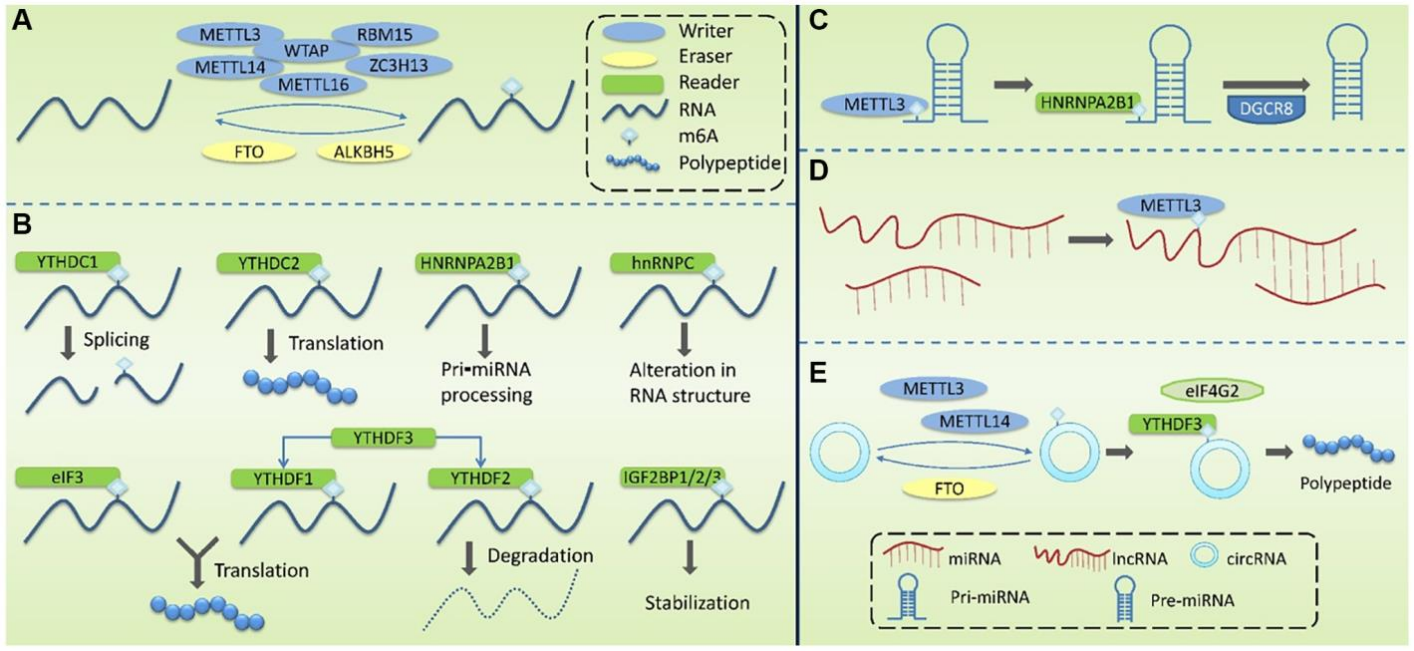
**Molecular mechanism associated with the modification of m6A methylation.** (**A**) m6A Writers are mainly composed of METTL3, METTL14, WTAP, METTL16, RBM15 and ZC3H13, mediating the modification of m6A methylation of RNA. Erasers mediate the process of m6A demethylation, mainly including FTO and ALKBH5. (**B**) m6A Readers recognize m6A methylation, including YTHDF1-3, YTHDC1-2, HNRNPC, HNRNPA2B1, eIF3, and IGF2BP1/2/3. (**C**) m6A modification plays a regulatory role in pri-miRNA to regulate the processing and maturation of miRNAs. (**D**) m6A modification plays a regulatory role in lncRNA to affect the RNA-RNA interaction function of lncRNA. (**E**) m6A modification plays a regulatory role in circRNA to promote its’ translation.

m6A methylation is a dynamic and reversible process [51], which is regulated by m6A methyltransferase complexes (m6A writers), m6A demethylases (m6A erasers), and m6A-binding proteins (m6A readers) (Figure 2A, 2B).

Writers take S-adenosylmethionine (SAM) as a methyl donor and mediate m6A methylation modification of RNA. These complexes are mainly composed of methyltransferase-like 3 (METTL3), methyltransferase-like 14 (METTL14), Wilms tumor1-associating protein (WTAP), methyltransferase-like 16 (METTL16), RNA binding motif protein 15 (RBM15), and zinc finger CCCH domain-containing protein 13 (ZC3H13). METTL3 contains a SAM-binding region [52] that identifies potential m6A modification sites and mediates the transfer of methyl from SAM to this site. METTL14 can form a heterodimer with METTL3 and specifically promote METTL3 recognizing RNA substrates. METTL3 and METTL14 are located on nuclear speckles and their localization is dependent on WTAP. By binding to the METTL3-METTL14 dimer, WTAP enables the methyltransferase complex to rapidly recognize potential m6A modification sites and activate the METTL3/METTL14 complex [53]. It has been reported that ZC3H13 can bind to WTAP and anchor the METTL3/METTL14 complex to the nucleus [54].

Erasers mediate the process of m6A demethylation, including the modulation of fat mass and obesity-associated gene (FTO) and AlkB homolog 5(ALKBH5). FTO oxidizes N-methyl at the m6A site to form hydroxymethyl groups [51, 55]. ALKBH5, co-locating with nuclear speaker in an RNaseA-sensitive manner, can directly catalyze the removal of methyl from 6-methylated adenosine and tends to demethylate specific m6A-modified single-strand RNA [56].

Readers recognize m6A methylation and act by specifically binding to the m6A-modified region or altering the RNA secondary structure to facilitate protein binding to RNA. Readers include YTH domain family (YTHDF) 1-3, YTH domain-containing proteins (YTHDC) 1-2, heterogeneous nuclear ribonucleoproteins (HNRNP), HNRNPC and HNRNPA2B1, eukaryotic translation initiation factor 3(eIF3), and insulin-like growth factor 2 mRNA-binding protein 1/2/3 (IGF2BP1/2/3). The role of YTHDF1-3 is mainly in the cytoplasm. YTHDF1 promotes the translation of m6A-labeled transcripts [57] and YTHDF2 promotes the degradation of m6A mRNA [58]. The combination of YTHDF3 with YTHDF1 enhanced its pro-translational ability, and the combination with YTHDF2 promoted its pro-degradation ability. The role of YTHDC1-2 is mainly in the nucleus. YTHDC1 may modulate pre-mRNA splicing factors to regulate RNA splicing [59], and YTHDC2 promotes mRNA translation [60]. The HNPNPC family regulates the selective splicing and structural alterations in mRNA [61]. HNRNPA2B1 interacts with the Di George Critical Region 8 (DGCR8) protein to promote pri-miRNA processing [62]. hnRNPC affects the local secondary structure of mRNA and lncRNA [61]. eIF3 binds to the m6A site in the 5′UTR of mRNA and promotes cap-independent mRNA translation [63]. IGF2BP1/2/3 increase the stability and translation efficiency of mRNA [64].

In recent years, m6A detection technology has developed rapidly, and a large number of m6A methylation sites have been identified by combining immunoprecipitation and high-throughput sequencing (meRIP-seq [65] and m6Aseq [66]). However, these two methods cannot identify m6A methylation sites that are very close to each other or accurately identify the m6A site. Photo-crosslinking co-immunoprecipitation techniques (such as PA-m6A-seq [67] and m6A-CLIP [68]) can achieve more accurate identification of m6A sites on the individual bases of RNA. In addition, the SCARLET method can detect single m6A sites with high accuracy [69]. However, it is expensive to detect m6A sites individually, and detection after bioinformatics prediction can greatly improve research efficiency. At present, multiple databases are available for researchers to use (Table 1), such as the WHISTLE database [70], SRAMP database (http://www.cuilab.cn/sramp/) [71], and HSM6AP database (http://lab.malab.cn/~lijing/HSM6AP.html) [72], which can be used to predict m6A sites. Verified m6A targets and potential m6A targets can be queried in the M6A2Target database (http://m6a2target.canceromics.org) [73], and WITMSG database (http://rnamd.com/intron/) can be used to predict m6A sites of introns [74]. m6A sites in specific cell lines or tissue types can be queried in the REPIC database (https://repicmod.uchicago.edu/repic) [75]. RMBase v2.0 database (http://rna.sysu.edu.cn/rmbase) [76] and iMRM (http://www.bioml.cn/XG_iRNA/home) [77] have also been used to predict methylation modification sites other than m6A. As m6A modification can be regulated by m6A regulatory proteins and affects a variety of physiological and pathological processes, databases integrating m6A functional annotations have emerged. RMBase v2.0 database (http://rna.sysu.edu.cn/rmbase) contains abundant information for exploring the relationship between RNA modification/miRNA binding and disease-associated single nucleotide polymorphisms (SNPs) [76]. Met-DB v2.0 database (http://compgenomics.utsa.edu/MeTDB/) integrates m6A “Writer,” “Eraser”, and “Reader” database, which facilitates the understanding of the function of m6A modification [78]. The development of technologies for m6A detection and prediction has greatly promoted in-depth research.

**Table 1:** m6A modification-associated databases.

**Database**	**Websites**	**Characteristic**	**Ref.**
WHISTLE		Predicted m6A sites	[70]
SRAMP	http://www.cuilab.cn/sramp/		[71]
HSM6AP	http://lab.malab.cn/~lijing/HSM6AP.html		[72]
M6A2Target	http://m6a2target.canceromics.org	Searched verified m6A targets and potential m6A targets	[73]
WITMSG	http://rnamd.com/intron/	Predicted m6A sites of introns	[74]
REPIC	https://repicmod.uchicago.edu/repic	Searched m6A sites in specific cell lines or tissue types	[75]
RMBase v2.0	http://rna.sysu.edu.cn/rmbase	a. Predicted methylation modification sites other than m6A b. Explored the relationship between RNA modification/miRNA binding and disease-associated SNPs	[76]
iMRM	http://www.bioml.cn/XG_iRNA/home	Predicted methylation modification sites other than m6A	[77]
Met-DB v2.0	http://compgenomics.utsa.edu/MeTDB/	Integrated m6A “Writer,” “Eraser”, and “Reader” database, which facilitates the understanding of the function of m6A modification	[78]

### Function and role of m6A RNA methylation

m6A modification occurs mainly in mRNA and it affects splicing, nuclear export, translation, and degradation of mRNA. Alterations in m6A modification can regulate mRNA processing (Table 2). FTO regulates exon splicing of the adipogenesis regulator RUNX1T1 by modulating m6A levels around the splicing site, thus regulating differentiation [79]. SR proteins are important regulators of alternative splicing. YTHDC1 promotes the RNA-binding ability of SRSF3 and inhibits that of SRSF10 to regulate mRNA splicing [59]. YTHDC1 also interacts with SRSF3 and RNA nuclear export factor 1 to regulate mRNA nuclear export [80]. The regulation of m6A modification in the degradation and translation of mature mRNA is another way to regulate gene expression. METTL3-mediated m6A modification of SOX2 mRNA increases transcription stability [81]. YTHDF1 [57] and IGF2BP1/2/3 are involved in regulating the translation of m6A-modified mRNA [64]. YTHDC2 enhances translation efficiency and reduces the stability of its target mRNA by interacting with translation and decay mechanisms [82]. It is reported that RNA m6A methyltransferase Mettl3 interacts with the 5′ external transcribed spacer (5′ETS) of the 47S rRNA precursor and modifies adenosine 196. Mettl3 knockdown results in the increase of pre-rRNA processing rates, while intracellular amounts of rRNA processing machinery components (U3, U8, U13, U14, and U17 small nucleolar RNA (snoRNA)and fibrillarin, nucleolin, Xrn2, and rrp9 proteins), rRNA degradation rates, and total amount of mature rRNA in the cell stay unchanged. Increased efficacy of pre-rRNA cleavage at A′ and A0 positions led to the decrease of 47S and 45S pre-rRNAs in the cell and increase of mature rRNA amount in the cytoplasm.

**Table 2:** Regulation of mRNAs by m6A modifications.

**Function**	**m6A regulatory proteins**	**Mechanism**	**Ref.**
mRNA processing	FTO	Regulate exon splicing of the adipogenesis regulator RUNX1T1 by modulating m6A levels around the splicing site, thus regulating differentiation	[79]
	YTHDC1	Promote the RNA-binding ability of SRSF3 and inhibit that of SRSF10 to regulate mRNA splicing	[59]
		Interact with SRSF3 and RNA nuclear export factor 1 to regulate mRNA nuclear export	[80]
Degradation and translation of mature mRNA	METTL3	Mediate m6A modification of SOX2 mRNA to increase transcription stability	[81]
	YTHDF1	Enhance translation efficiency of its target RNA	[57]
	IGF2BP1/2/3	Regulate the translation of m6A-modified mRNA	[64]
	YTHDC2	Enhance translation efficiency and reduce the stability of its target mRNA by interacting with translation and decay mechanisms	[82]

m6A is widely present in various cells and affects cell differentiation, apoptosis, and other biological processes, as well as tumor development and other pathological processes (Table 3). Cell differentiation is a process by which cells acquire different structure and function during ontogeny, and selective gene expression occurs during this stage. The regulation of m6A in mRNA enables m6A to regulate gene expression, thus affecting cell differentiation and ontogeny. There are multiple binding sites of METTL3 on the transcripts of pluripotent genes in mouse embryonic stem cells. It has been reported that METTL3 knockout decreases the m6A level of transcripts of pluripotent genes, which could inhibit the differentiation ability of embryonic stem cells [83]. FTO can downregulate the expression of ASB2 and RARA by reducing m6A levels in UTRs of transcripts, resulting in the inhibition of ATRA-mediated acute myeloid leukemia (AML) cell differentiation [84].

**Table 3:** Biological functions and role of m6A regulatory proteins in human diseases.

**Functions**	**m6A regulatory proteins**	**Mechanisms**	**Ref.**
Cell differentiation	METTL3	Binded to transcripts of pluripotent gene and regulate differentiation ability of embryonic stem cells	[83]
	FTO	Downregulated the expression of ASB2 and RARA by reducing m6A levels in UTRs of transcripts, resulting in the inhibition of ATRA-mediated AML cell differentiation	[84]
Spermatogenesis	METTL3	Altered splicing of spermatogenesis-related genes, thus regulating spermatogonial differentiation	[86]
	METTL3 and METTL14	Regulation of spermatogonial stem cells and spermatogenesis disorders	[85]
	ALKBH5	Regulated nuclear export of RNA and sperm malformation in mice	[87]
Central nervous system diseases	METTL3	Regulated mice cerebellar development	[88]
	METTL14	Regulated mice cerebral cortex development	[89]
	FTO	Regulated mice memory	[91]
Cardiovascular diseases	METTL3	Contributed to cardiac hypertrophy	[94]
		Promoted the maturation of miR-34a, which in turn inhibits SIRT1 and promotes the formation of abdominal aortic aneurysm	[96]
	FTO	Participated in reduced cardiomyocyte contractile function during heart failure	[95]
Tumors	METTL3	Promoted the maturation of miR-25-3p, thus activating the Akt-P70S6K pathway and promoting the initiation and development of PDAC	[97]
	FTO	Promoted glycolysis of breast cancer cells through the PI3K/AKT pathway	[98]
		Upregulated PKM2 expression through the demethylation of PKM2, thereby regulating glucose metabolism of HCC	[99]

m6A is closely related to spermatogenesis as well as the development and function of the central nervous system. It has been reported that the knockout of METTL3 and METTL14 results in the loss of spermatogonial stem cells and impaired spermatogenesis [85]. Moreover, METTL3 knockout causes decreased m6A levels and altered splicing of spermatogenesis-related genes, leading to the downregulation of gene expression and the regulation of spermatogonial differentiation [86]. A previous study reported that ALKBH5 deficiency in mice increased m6A levels and nuclear export of RNA, ultimately causing sperm malformation in mice [87]. During the development of the central nervous system, METTL3 knockout can lead to decreased m6A in the cerebellum of mice and increased apoptosis of new small brain cells, leading to severe cerebellar hypoplasia [88]. METTL14 knockout resulted in impaired cerebral cortex development in mice [89]. m6A modification in the brain is also involved in cognitive functions such as learning and memory [90]. Artificial deficiency of FTO in the dorsal hippocampus enhanced memory in mice [91]. Notably, abnormal m6A modifications can contribute to the development of Alzheimer’s disease [92]. In addition, m6A modifications can regulate the circadian rhythm of metabolism [93].

Abnormal regulation of m6A gene expression may promote the occurrence of certain diseases [90]. Changes in the expression of m6A play a regulatory role in the occurrence and development of cardiovascular diseases, such as cardiac hypertrophy, heart failure, and aortic aneurysm [91]. Hypertrophic stimulation leads to increased METTL3-mediated m6A modification, which contributes to cardiac hypertrophy [94]. In heart failure, FTO expression is reduced, leading to increased m6A levels and reduced cardiomyocyte contractile function. Decreased cardiomyocyte contractile function induced by ischemia can be alleviated by increasing FTO expression [95]. m6A modification promotes the maturation of miR-34a, which in turn inhibits SIRT1 expression and promotes the formation of abdominal aortic aneurysms [96]. The regulation of m6A modifications in tumors involves several aspects. Cigarette smoke induces the overexpression of METTL3 and upregulation of m6A in pri-miR-25 to promote its processing and maturation. miR-25-3p inhibits PHLPP2 expression, which in turn activates the Akt-P70S6K pathway and promotes the initiation and development of pancreatic ductal adenocarcinoma (PDAC) [97]. The energy metabolism of tumor cells differs from that of normal cells. Aerobic glycolysis promotes adaptation and rapid proliferation of tumor cells in the hypoxic microenvironment. FTO overexpression in breast cancer cell lines promotes glycolysis through the PI3K/AKT pathway [98]. FTO upregulates the expression of PKM2 through the demethylation of PKM2 mRNA in HCC, thus regulating glucose metabolism [99]. Abnormal modification of m6A in TME can modulate tumor immune escape. A previous study analyzed the mRNA expression profiles of 1,938 gastric cancer (GC) samples and established an m6A scoring system based on the status of 21 m6A regulators [100]. An increased mutation load and immune activation were observed with low m6A scores. Effective immune infiltration was not observed with a high m6A score. In fact, the modification of m6A is involved in the entire process of tumorigenesis and development, including the regulation of tumor cell proliferation, angiogenesis, metastasis and invasion, immune escape, and other aspects by promoting the formation of TME.

Aging is a natural process of body decline, characterized by decreased function of tissues and organs and increased risk of age-related diseases [15]. Brain aging is a complex process that affects the structural and functional connectivity of the brain. Morphologically, brain aging is characterized by volume loss, cortical thinning, white matter degeneration, loss of brain rotation, and ventricular enlargement. Pathologically, brain aging is related to neuronal cell atrophy, dendritic degeneration, demyelination, small-vessel disease, metabolic slowdown, microglia activation, and white matter lesions [19]. The mechanisms underlying these changes remain unclear, leading to a lack of effective therapies [23]. Epigenetic changes are considered to be important markers of aging and cellular senescence. Given the abundance and age-related changes in RNA m6A methylation in the CNS, it must play an important role in aging and degenerative neurological diseases. Altered m6A methylation modifications and mutated RNA m6A methyltransferases are associated with a variety of neuropathological processes, providing a new dimension for the study of brain aging.

### Regulation of ncRNAs by m6A modifications

m6A modification does not only occur in the ncRNA that can encode proteins, but also in miRNAs, and it plays an important regulatory role in the production and function of miRNAs (Figure 2C–2E; Table 4). ncRNA refers to functional RNA molecules that cannot be translated into proteins, including miRNAs, circRNAs, and lncRNAs.

**Table 4:** Regulation of ncRNAs by m6A modifications.

**ncRNA**	**m6A regulatory proteins**	**Mechanisms**	**Ref.**
miRNAs	METTL3	a. Marked pri-miRNA through m6A b. Increased Dicer splicing of pre-miRNAs	[103]
	HNRNPA2B1	Binded to m6A in pri-miRNA to recruit DGCR8, thereby promoting the maturation of miRNAs	[62]
lncRNAs	HNRNPC and HNRNPG	Binded RNA sequences surrounding m6A in lncRNA MALAT1	[61, 105]
	—	Played a role in the lncRNA-miRNA interaction, thereby influencing miRNA level	[106]
circRNAs	METTL3 and METTL14	Enhanced m6A-driven circRNA translation	[107]
	FTO	Inhibited m6A-driven circRNA translation	
	YTHDF3 and eIF4G2	Played key roles in m6A-driven circRNA translation	
	YTHDF2	Differentiated endogenous circRNA from exogenous circRNA and regulate innate immunity	[108, 109]

miRNAs are non-coding single-stranded small RNAs (18–24 nucleotides in length), which regulate gene expression at the post-transcriptional level through the formation of an RNA-induced silencing complex (RISC) [101]. The generation and maturation of miRNAs are regulated by m6A. In the nucleus, DNA is first transcribed into primary miRNAs (pri-miRNAs), which are then processed into precursor miRNAs (pre-miRNAs) by a microprocessor complex containing DGCR8 and DROSHA. Pre-miRNAs are then cleaved using Dicer into mature single-stranded miRNAs in the cytoplasm [102]. In this process, METTL3 marks pri-miRNA through m6A, and HNRNPA2B1 binds to m6A in pri-miRNA to recruit DGCR8; this enables DGCR8 to recognize and bind to specific substrates, thereby promoting the maturation of miRNAs [62, 103]. METTL3 can also promote miRNA biosynthesis by increasing Dicer splicing of pre-miRNAs.

lncRNAs are non-coding RNAs with more than 200 nucleotides that play a regulatory role in chromosomal inactivation and modification, transcription, shearing, translation, and protein activity. lncRNAs can also adsorb miRNAs through sequence complementation and inhibit the targeted regulatory effect of miRNAs on mRNA [104]. m6A modification in lncRNAs may influence RNA-protein interactions. m6A in lncRNA may make the surrounding RNA sequences more likely to bind HNRNPC and HNRNPG [61, 105]. It also plays a role in the lncRNA-miRNA interaction, thereby influencing miRNA level [106].

circRNA is formed by the covalent binding of the 3′ end and 5′ end of precursor mRNA after reverse splicing and can participate in biological processes, such as the regulation of miRNA expression and gene transcription. m6A can promote circRNA translation, and m6A in circRNA can act as an internal ribosomal entry site (IRES) for cap-independent translation [107]. METTL3 and METTL14 enhance m6A-driven circRNA translation, whereas FTO-mediated m6A demethylation inhibits this translation process, with YTHDF3 and eIF4G2 playing key roles in this process. In addition, m6A also plays a role in circRNA immunity. Endogenous circRNA binding to YTHDF2 cannot activate RIG-I, whereas exogenous circRNAs lacking m6A modification can activate the RIG-I pathway, leading to interferon production and induction of innate immunity [108, 109]. This indicates that m6A can differentiate endogenous circRNAs from exogenous circRNAs and regulate innate immunity.

### Regulation of m6A modifications by noncoding RNAs

**Figure 3:**
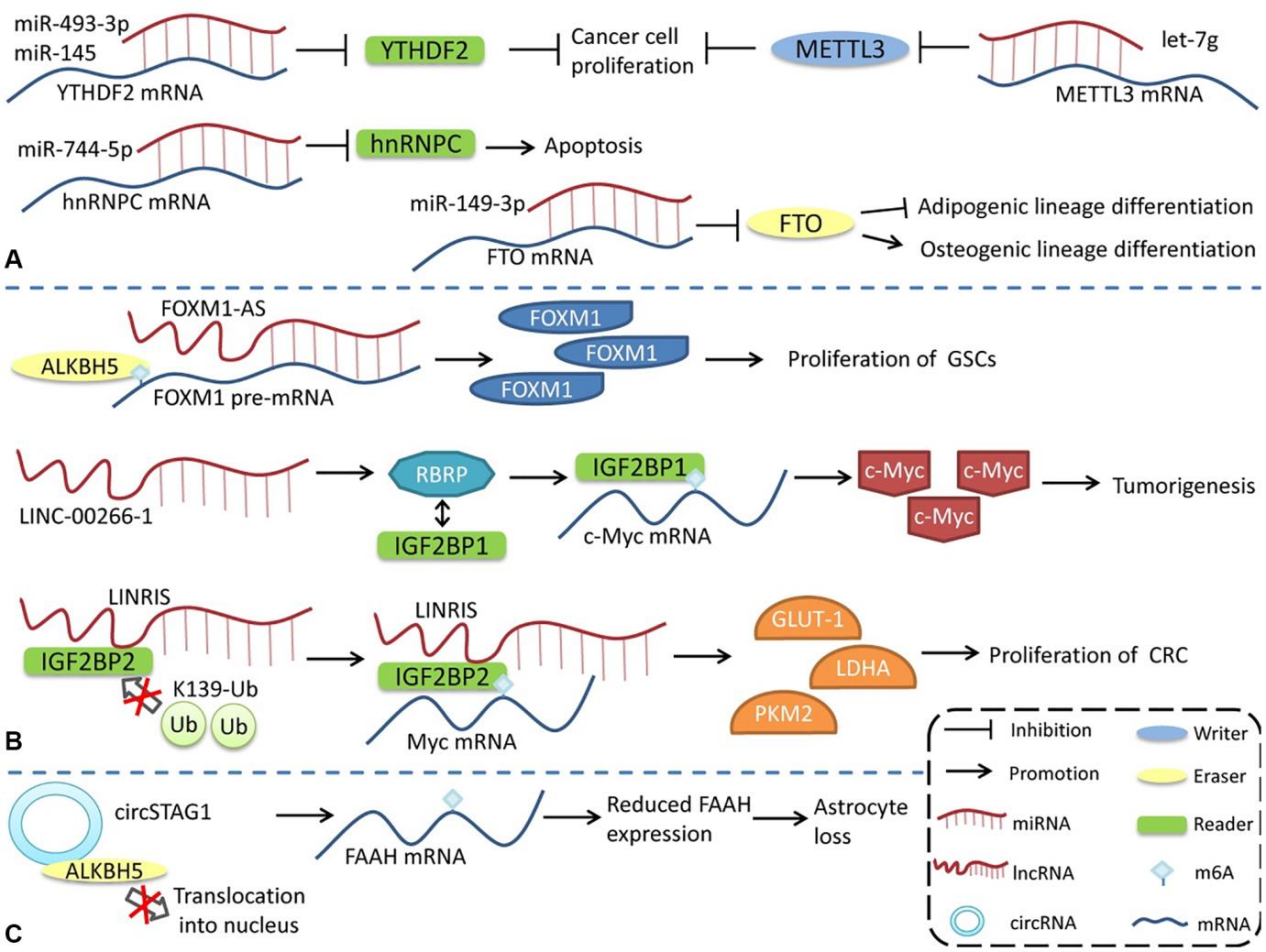
**Regulation of m6A modifications by ncRNAs.** ncRNAs can influence m6A modification by interacting with or regulating m6A regulatory proteins. (**A**) miRNA regulates the expression of m6A regulatory proteins. (**B**) lncRNA affects m6A modification. (**C**) circRNA interacts with m6A regulatory proteins to regulate m6A modification.

m6A modification can regulate the generation and function of ncRNAs. Conversely, ncRNAs, as important functional regulatory molecules, can also influence m6A modification by interacting with or regulating m6A regulatory proteins and participating in the regulation of various physiological and pathophysiological processes (Figure 3). miR-493-3p and miR-145 can downregulate YTHDF2 mRNA, alter the intracellular m6A level, and ultimately inhibit the proliferation of prostate [110] and liver cancer cells [111]. However, miR-744-5p can silence HNRNPC, which influences miR-21 expression and Akt phosphorylation and ultimately promotes apoptosis [112]. miR-149-3p binds to the 3′UTR of FTO mRNA to downregulate its expression, inhibit adipogenic lineage differentiation, and enhance osteogenic lineage differentiation [113]. miR-141 regulates IGF2BP2 in PDAC, and IGF2BP2 activates the PI3K-Akt pathway *in vivo* and promotes pancreatic cancer growth [114]. miRNA let-7g inhibits breast cancer development by targeting the 3′-UTR of METTL3 mRNA [115]. miR-600 can also downregulate METTL3 and inhibit the proliferation and migration of lung cancer cells [116].

In contrast to the way miRNAs directly bind to m6A mRNA to regulate its expression, lncRNAs modulates m6A by interacting with m6A regulatory proteins. LncRNA LINC-00266-1-encoded RBRP interacts with IGF2BP1 to enhance the recognition of m6A on c-myc mRNA, enhance the stability and expression of c-myc mRNA, and promote tumorigenesis [117]. The long intergenic non-coding RNA LINRIS maintains the stability of IGF2BP2 by blocking K139 ubiquitination and promoting colorectal cancer (CRC) proliferation [118]. LINC01234 interacts with HNRNPA2B1, leading to the recruitment of DGCR8 and promoting the processing and maturation of miR-106b-5p, which inhibits CRY2 and promotes the growth of non-small cell lung cancer (NSCLC) cells [119]. FOXM1-AS is an antisense lncRNA of FOXM1 that promotes the interaction of ALKBH5 with newborn FOXM1 transcripts, regulates m6A modification and FOXM1 expression, and regulates the proliferation of glioblastoma stem cell-like cells (GSCs) [120]. LncRNA GAS5-AS, the antisense lncRNA of GAS5, can also interact with ALKBH5 to adjust the m6A modification of GAS5 to enhance its stability [121].

The regulation of circRNAs by m6A modification has also been reported. circSTAG1 can bind ALKBH5, inhibit its entry into the nucleus, upregulate the m6A modification level of FAAH mRNA, and affect its stability and expression, resulting in astrocyte dysfunction [122]. However, compared with miRNA and lncRNA, the regulation of m6A modification by circRNA has a broader research scope. Notably, some m6A regulatory proteins have multiple functions, and the effects of m6A regulatory proteins are not necessarily entirely attributable to the alterations in the level of m6A modification. However, more detailed studies are needed to identify the role of different pathways in various physiological and pathological processes.

In addition, m6A modification can not only regulate the function of exosomes [123], but exosomal ncRNAs can also enter target cells and influence diseases occurrence by regulating the expression of m6A-related proteins [124–126]. Yuan et al. demonstrated that human umbilical cord mesenchymal stem cell (hucMSC)-derived exosomal miR-26a-5p can enter the nucleus pulposus (NP) cells to inhibit pyroptotic NP cell death by targeting the METTL14/NLRP3 axis, thereby suppressing the progression of intervertebral disc degeneration (IVDD) [124].

## Role of m6A modification and ncRNAs in TME

**Figure 4:**
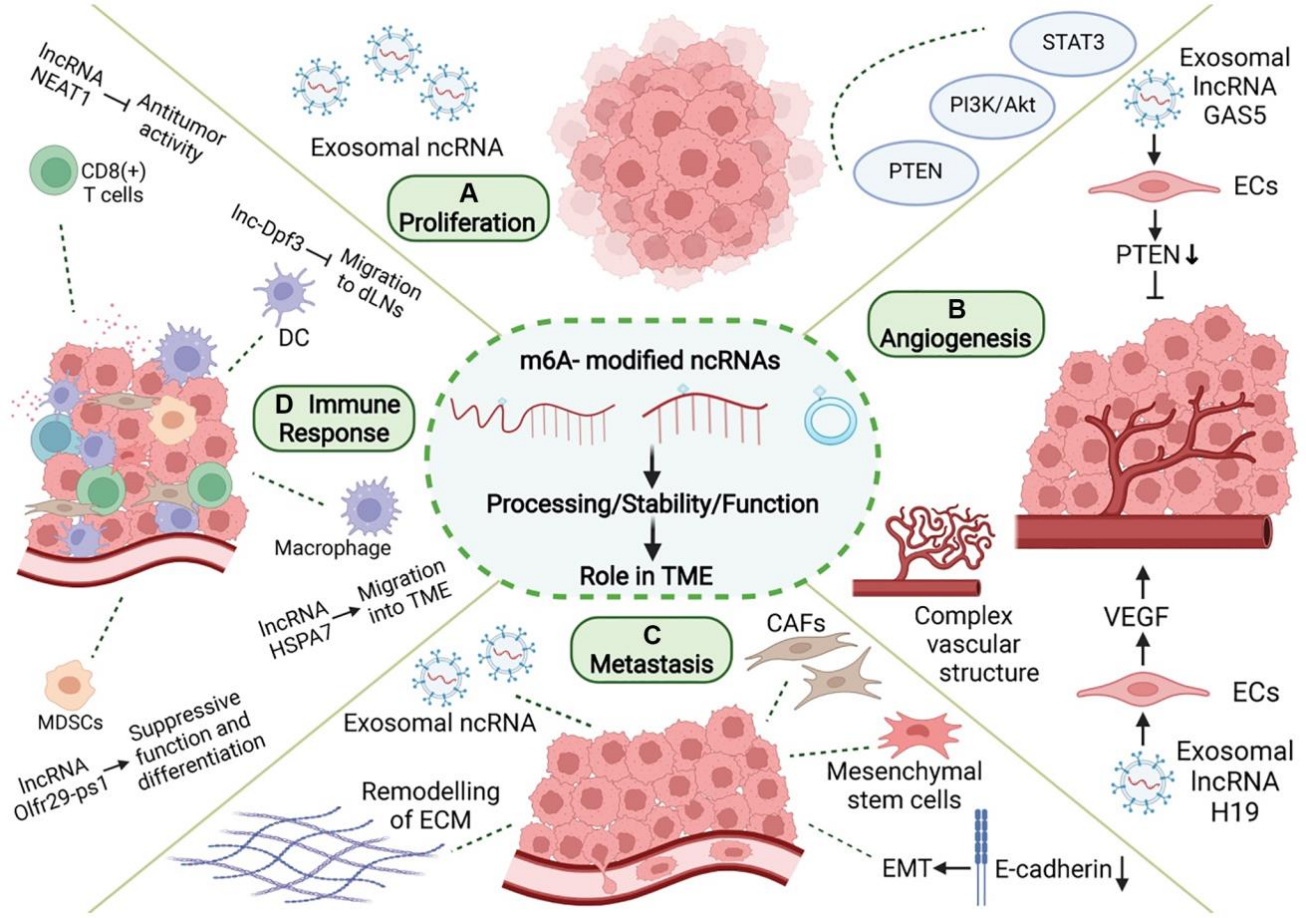
**Role of m6A modification and ncRNAs in TME.** In TME, m6A-related ncRNAs are involved in the regulation of proliferation, angiogenesis, invasion and metastasis, and immune escape in tumor. (**A**) m6A-related ncRNAs can affect tumor proliferation by regulating proliferation-related genes and signaling pathways, and can be transmitted in TME through exosomes. (**B**) m6A-related ncRNAs regulate tumor angiogenesis by regulating pro-angiogenic molecules, such as VEGF, and are involved in angiogenesis patterns of large vessels and complex vascular structures. (**C**) Tumor metastasis is related to EMT in tumor cells and the dissolution and remodeling of ECM. m6A-associated ncRNAs can also be delivered through exosomes, while regulating stromal cells in TME to promote metastasis. (**D**) m6A-related ncRNAs are involved in the recruitment, differentiation, and functional expression of immune cells in TME, and they promote the immune escape of tumor cells.

During tumorigenesis, tumor cells interact closely with stromal cells and immune cells in the microenvironment, jointly transforming the microenvironment into a site conducive to the growth, invasion, and metastasis of tumor. m6A-related ncRNA, as an important means of intracellular regulation of gene expression and functional status, as well as intercellular communication, can promote the formation and maturation of TME and play an important role in proliferation, angiogenesis, invasion, metastasis, immune escape, and other processes in tumor (Figure 4).

### Mediation of tumor proliferation

Cell proliferation involves complex signal transduction. Uncontrolled proliferation of tumor cells is mostly related to the abnormality of proliferation-related genes and impaired signaling pathways involving cellular processes, such as oncogene activation, tumor suppressor gene inactivation, apoptosis resistance, and metabolic reprogramming [127, 128]. However, the malignant proliferation of tumors is related to tumor cells, and TME can also enhance this abnormal proliferation. In various malignant tumors, CAF in TME can promote tumor proliferation through the synthesis and secretion of TGF-β, fibroblast secretory protein 1 (FSP1), SDF-1, and other growth factors [129]. Mast cells can promote tumor proliferation through direct contact with tumor cells or the secretion of factors such as IL-17A [130].

m6A-related ncRNAs also play a regulatory role in tumor proliferation. m6A modification of ncRNAs can influence the expression of ncRNA by changing its stability or maturation process and regulating the expression of proliferation-related genes or signal pathways, thus promoting or inhibiting tumor proliferation. METTL3 promotes m6A-dependent miR-221/222 maturation and downregulates PTEN, thereby promoting the proliferation of bladder cancer cells [131]. Deoxycholic acid (DCA) levels are significantly decreased in gallbladder cancer (GBC) [132]. DCA can reduce the m6A level of pri-miR-92b, thereby reducing its expression and upregulating PTEN and consequently inhibiting tumor proliferation mediated by the PI3K/AKT pathway [132]. Moreover, m6A-related ncRNAs also play an important regulatory role in TME, which may serve as a bridge between tumor proliferation and TME formation. The signals of abnormal proliferation in a single tumor cell may also be transmitted to other tumor cells by m6A-associated ncRNAs through TME, thereby promoting the spread of malignant phenotypes. The links between these three factors have not been elucidated. ALKBH5 can mediate m6A demethylation of lncRNA PVT1 in osteosarcoma, thus upregulating PVT1 and promoting osteosarcoma cell proliferation [133]. PVT1 can also promote the transport and fusion of multivesicular bodies (MVB) to the plasma membrane, thus promoting exosome secretion by pancreatic cancer cells [134]. Exosomes are important intercellular communication molecules that can promote information transmission between tumor cells, and between tumor cells and the tumor matrix, thus promoting the formation of TME and tumor development. These results suggest that m6A-related ncRNAs may be involved in tumor development by regulating the formation of tumor cells and TME. IGF2BP2 promotes the stability and expression of ZFAS1 in an m6A-dependent manner [135]. In esophageal squamous cell carcinoma (ESCC), ZFAS1 can shuttle between tumor cells in the form of exosomes and act as ceRNA to downregulate miR-124, thereby upregulating STAT3 and ultimately inhibiting cellular apoptosis and promoting proliferation, migration, and invasion [136]. These results suggest that m6A-related ncRNA-mediated tumor proliferation may spread through exosomes in TME and increase tumor malignancy. In conclusion, tumor cells are closely related to other components of TME, and m6A-related ncRNAs can simultaneously regulate tumor proliferation and TME. Further studies are needed to confirm the potential crosstalk between the two factors.

### Mediation of tumor angiogenesis

Tumor tissues require abundant blood flow to provide nutrients and excrete metabolites. In the process of tumor tissue growth and enlargement, the normal blood vessels of the original tissue are insufficient to support further growth. At this time, the components of TME can establish new blood circulation in the tumor tissue through complex signal communication. The rapid growth of tumor cells leads to a local anoxic microenvironment with metabolite accumulation, which promotes the formation of angiogenic mimicry and increases blood supply by promoting ECM remodeling and inducing the transformation of cancer stem cells (CSC) into endothelial phenotypes [137]. In addition, CAFs and TAMs in TME can also secrete angiogenic factors, such as VEGF, and cytokines, such as interleukin and free ncRNAs, into TME to activate the angiogenesis signaling pathway and promote tumor angiogenesis [138, 139].

m6A-related ncRNA is one of the components released by tumor cells into TME to promote angiogenesis and plays a regulatory role in the tumor angiogenesis microenvironment. MiR-155 can target FTO and upregulate m6A levels in clear cell renal cell carcinoma (RCC) [140]. However, melanoma-derived exosome miR-155 can be delivered to fibroblasts to downregulate SOCS1 and then activate the JAK2/STAT3 pathway, leading to upregulated VEGFa, FGF2, and MMP9 in the fibroblasts, enhanced reprogramming of fibroblasts into CAF, and improved angiogenesis promotion capacity [141]. m6A modification can regulate the expression of H19 and miRNA675 [142]. H19 is the precursor lncRNA of miRNA675, both of which regulate angiogenesis. MiRNA675-5p is involved in angiogenesis in anoxic microenvironments [143]. H19 is abundant in the exosomes secreted by CD90+ HCC cells. Through the adhesion of CD90 to human umbilical vein endothelial cells (HUVECs), exosomes can invade endothelial cells, deliver H19 to target cells, and stimulate angiogenesis through the synthesis and release of VEGF [144]. VIRMA is a regulatory component of m6A, and its knockout can reduce the stability and expression of lncRNA CCAT1 through decreased m6A level [145]. Pancreatic cancer (PC) cell-derived exosomes can transport CCAT1 to HUVEC. CCAT1 upregulates HMGA1 expression through competitive binding of miR-138-5p and enhances angiogenesis in HUVEC [146]. YTHDF3 promotes the degradation of lncRNA GAS5 in an m6A-dependent manner [147]. GAS5 is expressed at low levels in human lung cancer tissues, lung cancer cells, and cell culture supernatant exosomes. The overexpression of GAS5 in lung cancer cells can inhibit proliferation and tube formation in HUVECs in the form of exosomes, thereby affecting angiogenesis. The mechanism involves the regulation of PTEN expression, as well as PI3K and AKT phosphorylation through the “sponging” of miR-29-3p [148]. Therefore, tumor cells can increase the levels of pro-angiogenic m6A-related ncRNAs and decrease those of the anti-angiogenic m6A-related ncRNAs in secreted exosomes, thus regulating the angiogenesis process in TME. This provides a new research idea for anti-angiogenesis in tumor research. m6A modification of pri-miR-17-92 promotes its processing and maturation [149]. MiR-20a, a member of the miR-17-92 cluster, is associated with angiogenesis patterns in large vessels and complex vascular structures in breast cancer [150]. Tumor angiogenesis is an important event in TME, and changes in angiogenesis patterns may affect nutrient supply to various cells in TME, thus affecting their functional status. In addition, m6A modification can promote the splicing of the precursor miR-143-3p to promote its maturation. MiR-143-3p increases VEGFA expression in lung cancer cells by downregulating VASH1 and promoting angiogenesis [151]. miR-143-3p is highly expressed in the EVs of osteosarcoma cells and involved in the regulation of TME [152]. The specific role of miR-143-3p in TME is still unclear, and further research is needed to determine if miR-143-3p can also affect tumor angiogenesis by regulating TME. In conclusion, the multiple roles of m6A-related ncRNAs in TME may involve a regulatory network that modulates angiogenesis and tumor progression through multiple pathways.

### Mediation of tumor metastasis

Tumor metastasis is closely related to EMT as well as the adhesion, invasion, and migration of tumor cells [153, 154]. The reduction in adhesion molecules on the surface of tumor cells allows cells to separate from each other, facilitating cell mobility. The ECM is a key regulator of tumor invasion and metastasis. Tumor or stromal cells can produce proteases (such as MMP) to dissolve ECM components and promote tumor cell migration. EMT is the transformation of tumor cells from an epithelial to a more aggressive mesenchymal phenotype, which promotes tumor metastasis. During EMT, E-cadherin and other cell adhesion proteins are gradually reduced, while N-cadherin, vimentin, and other mesenchymal marker proteins are enriched. Tumor metastasis is closely related to the enhanced invasion and metastatic ability of tumor cells as well as changes in TME. TME can promote tumor metastasis by promoting EMT and the invasion ability of tumor cells, promoting ECM remodeling and the formation of a premetastatic niche [155].

m6A-related ncRNAs play a significant regulatory role in many aspects of tumor metastasis. First, m6A-related ncRNAs can influence tumor invasion and metastasis by regulating the expression of metastasis-related genes. m6A modification can promote the splicing and maturation of miR-143-3p, while miR-143-3p can downregulate E-Cadherin and upregulate FN, vimetin, MMP2, and MMP-9 in lung cancer cells to promote the occurrence of EMT [151]. In nasopharyngeal carcinoma, m6A modification can enhance the stability of lncRNA FAM225A. As ceRNA, FAM225A inhibits the expression of miR-590-3p and miR-1275 and upregulates downstream ITGB3, promoting proliferation, migration, and invasion of cancer cells through the FAK/PI3K/Akt pathway [156]. In addition, future studies need to investigate if m6A-related ncRNAs regulate tumor metastasis by modulating TME. Small extracellular vesicles (sEV) derived from gastric cancer (GC) containing miR-151a-3p can be absorbed by hepatic Kupffer cells (KCs), and sEV-miR-151a-3p targets YTHDF3 and downregulates SUMO1 translation in an m6A-dependent manner, thereby inhibiting SP3, inducing TGF-β1 expression, and promoting the formation of a local liver metastasis microenvironment through the activation of the SMAD2/3 pathway [157]. METTL3 promotes the maturation of pri-miR-320b in an m6A-dependent manner, and miR-320b promotes EMT by downregulating PDCD4 and can be included in exosomes to promote lymphangiogenesis, thereby promoting ESCC metastasis [158]. LncRNA XIST is misexpressed in a variety of tumors and can regulate the malignant phenotype of tumors. YTHDF2 recognizes m6A modifications in XIST and mediates their degradation [159]. The deletion of XIST in breast cancer can promote the phenotypic transformation of microglia through the transport of miR-503 exosomes to microglia, leading to local immunosuppression and promoting brain metastasis in breast cancer [160]. ALKBH5 can upregulate NEAT1 by mediating m6A demethylation, thereby influencing the expression of EZH2 and inhibiting the invasion and metastasis of GC cells [161]. Bone metastases from prostate cancer are typically osteogenic. NEAT1 can be transferred to human bone marrow-derived mesenchymal stem cells (hBMSCs) through exosomes secreted by prostate cancer cells, thereby upregulating RUNX2, promoting osteogenic differentiation [162], and participating in the formation of the local bone metastasis microenvironment. METTL3 and YTHDF3 increase the stability of MALAT1 in an m6A-dependent manner, and MALAT1 acts as ceRNA to “sponge” miR-1914-3p, thereby upregulating YAP and promoting NSCLC invasion and metastasis [163]. In a GC study, MALAT1 increased the accumulation of SQSTM1 in tumor cells, which in turn activated NF-κB and increased the expression of IL-6. IL-6 promotes paracrine transformation of CAF [164]. CAF secrete various cytokines to promote tumor metastasis. This suggests that m6A-related ncRNAs can not only change the gene expression of tumor cells to enhance their ability to invade and metastasize but also promote tumor metastasis by inducing the formation of TME. Tumor cells and TME carry out complex information transmission to synergistically promote tumor invasion and metastasis, and m6A-related ncRNAs play an important mediating role in this process. However, further research is needed to gain clearer understanding of the regulatory network of TME in different tumor types.

### Mediation of tumor immune response

Under normal circumstances, the immune surveillance function of the immune system can promptly detect and eliminate tumor cells caused by gene mutations; however, the formation of TME can protect tumor cells from the immune system. In TME, the recruitment, differentiation, and functional expression of immune cells are precisely regulated, forming a low anti-tumor immune response microenvironment to promote the immune escape of tumor cells. m6A modification plays an important role in maintaining the normal function of immune cells, and changes in m6A modification can affect the formation of an immunosuppressive microenvironment in tumors [165]. In DC, METTL3-mediated m6A modification promotes the expression of the costimulatory molecules, CD40 and CD80, and the proinflammatory cytokine IL-12, and promotes DC activation and maturation [166]. YTHDF1 recognizes the m6A modification of lysosomal protease transcription and promotes its expression, which in turn blocks cross-presentation of tumor antigens by DC and antigen-specific activation of CD8+ T cells [167]. METTL3 can also improve the stability of STAT1 mRNA by mediating m6A modifications, upregulating STAT1, and driving the polarization of M1 macrophages [168]. FTO knockout inhibits NF-κB signaling and decreases the stability of STAT1 and PPAR-γ mRNA, thus impeding macrophage activation [169]. In mouse models, METTL3 deficiency upregulated the mRNA expression of SOCS family by modulating mRNA m6A modification, thus inhibiting IL-7-mediated STAT5 activity and the homeostasis, proliferation, and differentiation of T cells [170]. METTL14/YTHDF1 knockdown in GC cells downregulated IFN-α, -β, and-γ transcription levels [171]. As m6A modification is ubiquitous in cells, its changes often involve alterations in multiple genes and signaling pathways, providing clues for subsequent in-depth studies.

m6A-related ncRNAs can regulate the migration, differentiation, and functional states of various immune cells, making the regulatory network of the tumor immune microenvironment more detailed and precise. The lncRNA Olfr29-ps1 can interact with miR-214-3p to inhibit its expression, thereby promoting the immunosuppressive function and differentiation of mononuclear MDSCs. In GM-CSF + IL6-induced MDSCs, Olfr29-ps1 was modified by m6A, which enhanced the regulation of Olfr29-ps1 on MDSCs [172]. The lncRNA HSPA7 is significantly overexpressed in glioblastoma (GBM) tissues, and m6A modification of HSPA7 promotes its expression. HSPA7 promotes macrophage migration to the GBM TME by activating the YAP1-LOX axis and can promote an immunosuppressive phenotype and inhibit the anti-tumor immune response [173]. In colorectal cancer, METTL3 upregulated the expression of miR-1246 by promoting the maturation of pri-miR-1246 through methylation [174]. However, exosomes shed by mutant p53 colon cancer can carry miR-1246 to the microenvironment containing macrophages, where it reprograms macrophages and recruits immunosuppressive T cells to promote tumor development [175]. DC migration to draining lymph nodes (DLNs) plays an important role in the initiation of anti-tumor T cells and adaptive immunity. CCR7 activation can prevent lnc-Dpf3 degradation by removing its m6A modification. Lnc-Dpf3 is upregulated after recognition by YTHDF2. Upregulated lnc-Dpf3 can directly bind to HIF-1α protein and inhibit the transcription of Ldha, an HIF-1α dependent glycolysis gene, thus inhibiting glycolysis metabolism in DCs and CC7-mediated DC migration to DLNs, which plays an immunosuppressive role in tumors [176]. In GC, ALKBH5 can upregulate lncRNA NEAT1 through demethylation [161]. In HCC, the downregulation of NEAT1 can enhance the anti-tumor function of CD8+ T cells [177]. m6A modification of circIGF2BP3 upregulates its expression by promoting its reverse splicing and cyclization. circIGF2BP3 upregulates PKP3 through competitive binding of miR-328-3p and miR-3173-5p, thereby inhibiting PD-L1 ubiquitination and promoting the immune escape of NSCLC cells [178]. As immunotherapy has become a widely used anti-tumor therapy, these studies undoubtedly provide a new research direction for precise tumor therapy.

## Potential clinical application of m6A-modified ncRNAs in cancers

### m6A-related ncRNAs as potential biomarker

m6A-related ncRNAs are closely related to tumorigenesis and development, which makes them a biomarker for tumor diagnosis and prognosis evaluation and a new target for the development of anti-tumor drugs. m6A-related regulatory proteins and the level of m6A modification in peripheral blood have shown potential as tumor biomarkers. The level of RNA m6A modification in the peripheral blood of patients with GC can be used as a marker for GC screening, and the diagnostic value of m6A can be improved by combining it with other tumor markers, such as CEA or m6A demethylases, such as ALKBH5 and FTO. The downregulation of m6A after surgery makes it a possible marker for follow-up [179]. YTHDF3 and VIRMA are significantly overexpressed in testicular seminoma and may be new markers for the identification of testicular germ cell tumor subtypes [180].

However, m6A regulatory proteins often simultaneously regulate the m6A modification of multiple target RNAs, and different target RNAs play different roles in tumor cells. Therefore, it may not be accurate enough to use m6A regulatory proteins as biomarkers to screen for or predict tumor prognosis. m6A-associated ncRNAs may represent a new class of tumor markers (Table 5). For example, YTHDC1 promotes the export of circNSUN2 from the nucleus to the cytoplasm in an m6A-dependent manner. In CRC, circRNA circNSUN2 is upregulated in the serum and metastatic liver tissue of patients with liver metastasis, and it is associated with poor prognosis [181]. m6A level of lncRNA NEAT1-1 is a poor prognostic factor for prostate cancer, and high m6A levels are associated with bone metastasis [182]. m6A modification can enhance the stability of lncRNA LNCAROD, and LNCAROD is associated with shortened overall survival of head and neck squamous cell carcinoma (HNSCC) [183]. In epithelial ovarian cancer (EOC), m6A modification upregulates lncRNA RHPN1-AS1 by increasing its transcriptional stability, whereas high RHPN1-AS1 levels are significantly associated with distant metastasis and death [184]. Combining multiple m6A-related ncRNAs to build risk models provides guidance for clinicians in making improved diagnosis and treatment decisions. A new prognostic index, m6AlRsPI, was constructed based on two m6A-modified hub lncRNAs in kidney renal clear cell carcinoma (KIRC). High m6AlRsPI is associated with poor prognosis, and its area under the ROC curve (AUC) for predicting 3-year and 5-year survival is 0.760 and 0.677, respectively [185]. In metastatic cutaneous melanoma, an m6A-associated lncRNA model (m6A-LncM) containing 24 lncRNAs was constructed, most of which had potential m6A modification sites. The AUC for predicting 3-year, 5-year, and 10-year survival were 0.778, 0.813, and 0.828, respectively [186]. m6A-LPS, which is composed of 12 m6A-associated lncRNAs, is an independent prognostic factor of breast cancer, with an AUC of 0.776, and these 12 lncRNAs are associated with clinicopathological features, such as age, sex, as well as T, M, and N stage of patients with breast cancer [187].

**Table 5:** Correlation between m6A-related ncRNAs and clinical pathological characterizations of tumor patients.

**Tumor types**	**Sample type**	**m6A-related ncRNAs**	**Expression**	**Biomarker type**	**Ref.**
CRC	Serum and tissues	circNSUN2	Upregulated	CircNSUN2 is associated with poor prognosis.	[181]
Prostate cancer	Tissues	lncRNA NEAT1-1	Upregulated	High m6A levels are associated with bone metastasis.	[182]
HNSCC	Tissues	lncRNA LNCARD	Upregulated	LNCAROD is associated with shortened overall survival of HNSCC.	[183]
EOC	Tissues	lncRNA RHPN1-AS1	Upregulated	High RHPN1-AS1 levels are significantly associated with distant metastasis and death	[184]
KIRC	Tissues	LINC01820 and LINC02257	Upregulated	High levels are associated with poor prognosis.	[185]
Pancreatic cancer	Serum	Serum miR-17-5p m6A level	Methylation level increased	Serum miR-17-5p m6A level is a potential marker for the early diagnosis of pancreatic cancer.	[188]
Glioma	Tissues	hsa_circ_0127664 and hsa_circ_0008362	Methylation level increased	hsa_circ_0127664 and hsa_circ_0008362 could be used as potential diagnostic markers.	[189]
NSCLC	Tissues	Exsomal miR-4443	Upregulated in CIS-R NSCLC	miR-4443 is expected to be a marker of cisplatin response in NSCLC.	[125]

m6A-related ncRNAs can be used to predict tumor prognosis and also serve as markers for the early diagnosis of tumors and markers related to the tumor immune microenvironment. Serum miR-17-5p methylation levels increase in patients with early pancreatic cancer, compared with healthy controls, which makes it a potential marker for the early diagnosis of pancreatic cancer [188]. The AUC of hsa_circ_0127664 was 0.8044 in diagnostic tests of patients with high-grade glioma (HGG) and low-grade glioma (LGG). The AUC of hsa_circ_0008362 in the diagnostic tests of HGG and normal tissues was 0.9467, suggesting that these circRNAs could be used as potential diagnostic markers. Hsa_circ_0127664 and hsa_circ_0008362 have relatively high levels of m6A modification, but their specific significance is unclear [189]. m6A-related ncRNAs have also shown potential in the large-scale detection of cancer, and a serum m6A-miRNAs diagnostic signature has high accuracy and sensitivity, which should make it a low-cost, minimally invasive novel biomarker [190]. In addition, because m6A-related ncRNAs are closely related to TME and participate in the formation and dynamic regulation of TME, some m6A-related ncRNAs are expected to become potential markers or therapeutic targets related to TME, opening a new path for the accurate diagnosis and effective treatment of tumors. Some studies constructed an m6A-related ncRNA model and combined it with the analysis of immune cells, immune-related molecules, or pathways, and some m6A-related lncRNAs that may be associated with the tumor immune microenvironment were predicted in colorectal adenocarcinoma [191], lung adenocarcinoma [192], renal clear cell carcinoma [193] and breast cancer [187], which can be used to predict immunotherapy response. Several m6A-associated lncRNAs, including LINC00342, are positively correlated with PD-1 expression in renal clear cell carcinoma [193], which could be a potential target for enhanced efficacy of immune checkpoint inhibitors against PD-1. As one of the important mediators of signal transduction in TME, m6A-related ncRNAs in exosomes are also expected to become markers of TME. MiR-4443 targets METTL3 in NSCLC, thereby upregulating FSP1 expression in an m6A-dependent manner, inhibiting cisplatin-induced iron death, and promoting tumor proliferation. Cisplatin-resistant (CIS-R) tumors release exosomes containing miR-4443, which are transferred to cisplatin-sensitive (CIS-S) cells to confer cisplatin resistance. The expression level of exosomal miR-4443 in CIS-S NSCLC is approximately one-third of that in normal lung tissue, while that in CIS-R NSCLC is approximately 1.5-fold higher than that in normal lung tissue. Therefore, miR-4443 is expected to be a marker of cisplatin response in NSCLC [125].

### m6A-modified ncRNAs as therapeutic targets

The important role of m6A-related ncRNAs in TME makes them a new target for anti-tumor therapy. Because m6A methylation and demethylation are reversible processes, it is possible to change the expression of m6A-modified ncRNA by targeting m6A regulatory proteins, thus exerting an anti-tumor therapeutic effect. R-2HG can inhibit FTO by increasing m6A levels and suppressing leukemia cell proliferation. R-2HG combined with chemotherapy had a synergistic anticancer effect in a mouse leukemia model [194]. Meclofenamic acid is another FTO inhibitor [195], and its derivative, MA2, upregulates m6A levels in glioblastoma stem cells. In a glioblastoma mouse model, intratumoral injection of MA2 inhibited tumor growth and extended survival [196]. However, the unique advantages and disadvantages of ncRNA in animal and clinical trials can provide a reference for the development of m6A-related ncRNA-targeted drugs. The knockdown of lncRNA MALAT1 using an antisense oligonucleotide technique in a mouse breast cancer model resulted in decreased tumor growth and reduced metastasis [197]. MRX34 is an analog of tumor suppressor miR-34a and significantly inhibits tumor growth in mouse models [198]. In phase I clinical trials in patients with advanced solid tumors, partial responses (PR) or stable disease (SD) were achieved in some patients [199, 200], but further studies are needed to reduce immune-related toxicity of miRNA [200]. Intratumoral injection of circNRIP1 siRNA inhibited tumor growth in a GC mouse model [201]. In addition, a circRNA vaccine against SARS-COV-2 induced effective immune responses in both mouse and monkey models [202]. However, it is not clear if circRNA vaccines can be used to induce anti-tumor immunity.

**Figure 5:**
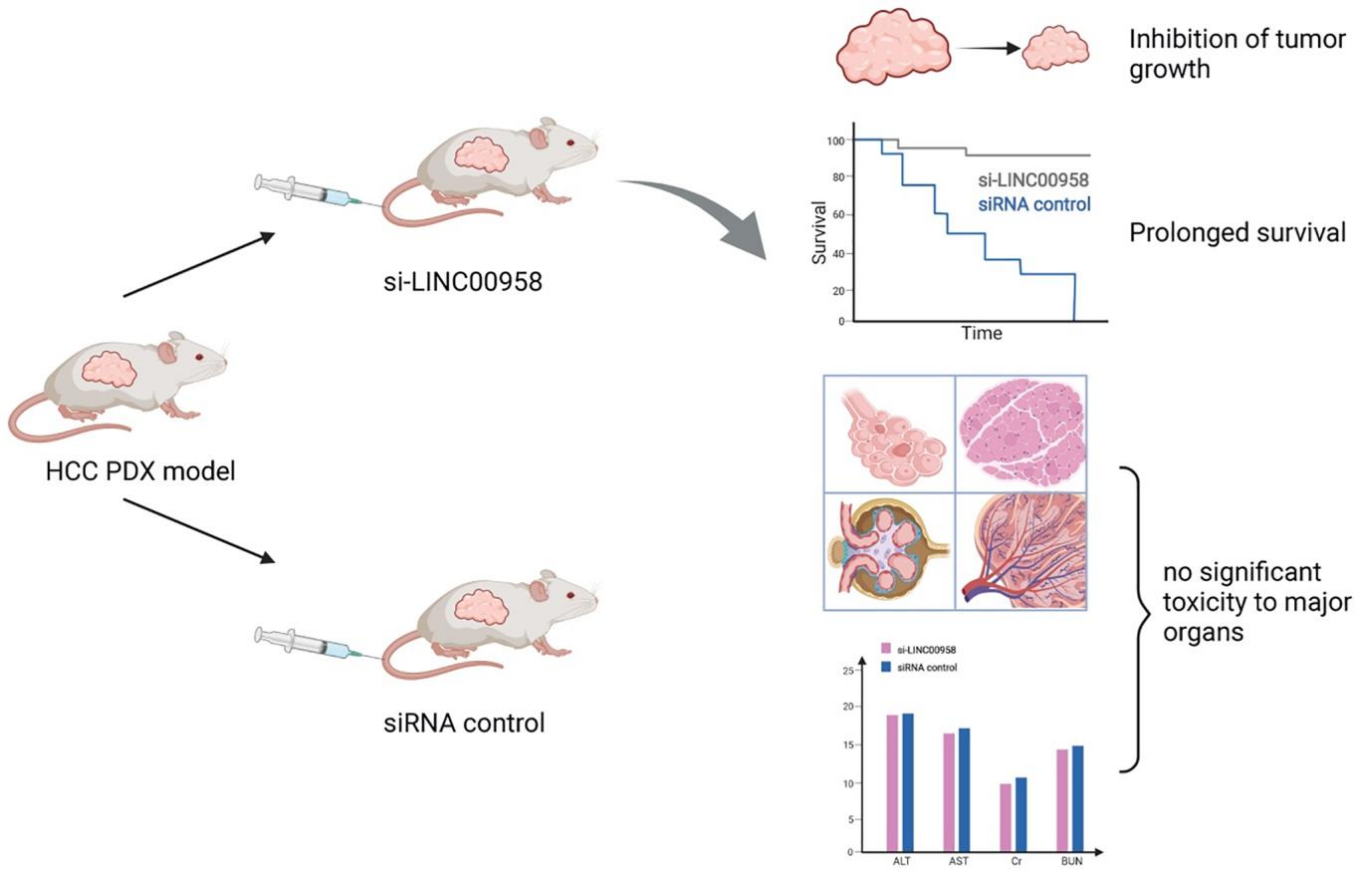
**Schematic diagram of a patient-derived xenograft mouse model of liver cancer treated with si-LINC00958.** A drug system loaded with si-LINC00958 effectively inhibited tumor growth in the PDX mouse model and significantly prolonged the overall survival of the mice. The systemic toxicity of the drug was evaluated using hematoxylin and eosin staining and blood indexes, and there was no evident toxicity to the liver, kidney, lung, spleen, and heart.

The regulation of m6A-related ncRNAs in TME makes them a potential therapeutic target for tumor growth inhibition and enhanced sensitivity to chemotherapy. m6A modification can upregulate LINC00958 by enhancing its stability, and LINC00958 can promote the proliferation, migration, invasion, and adipogenesis of HCC through the miR-3619-5p/HDGF axis. PEGylated PLGA nanoparticles loaded with si-LINC00958 effectively inhibited tumor growth in a patient-derived xenograft (PDX) mouse model, significantly prolonged the overall survival of the mice, and showed no significant toxicity to the liver, kidney, lung, spleen, or heart [203] (Figure 5). The upregulation of m6A levels at specific sites of circRNA-SORE in HCC cells can enhance RNA stability, lead to upregulation of circRNA-SORE, and ultimately promote sorafenib resistance in cancer cells. In animal models, the local injection of circRNA-SORE interfering RNA significantly improved the efficacy of sorafenib [204]. High-risk human papillomavirus produces m6A-modified circRNA circE7, which translates into the E7 oncoprotein and promotes cell transformation. After circE7 knockdown by shRNA, tumor growth was inhibited both *in vitro* and in a xenograft, mouse model [205]. The practical application value of m6A-related ncRNAs as anti-tumor therapeutic targets remains to be further explored.

### Hypoxia and m6A modification

Excessive distance between the vascular system and tumor cells can lead to diffuse hypoxia [93]. Under the condition of excessive tissue hypoxia, the homeostasis of the microenvironment is destroyed, resulting in hypoxia, hypoglycemia and acidic TME, which is conducive to tumor growth. Notably, hypoxia and tumor growth form a mutually positive feedback loop. Tumor cell proliferation leads to excessive oxygen depletion in Tmes and promotes anoxic environment in Tmes, thus providing suitable conditions for the occurrence and metastasis of tumors through proliferation, differentiation, drug resistance and other ways [116]. Cell hypoxia is mainly controlled by hypoxia-inducible factors (HIF), and heterodimer helix-loop-helix proteins composed of O2-unstable α and constitutive expressed β subunits (HIF1α, HIF2α and HIF-1β) participate in the coordination and regulation of many mechanisms to adapt tumor cells to the harsh environment [123]. HIF-1α and HIF-2α mainly recognize similar hypoxia response elements in target gene promoters and play a stable role in anoxic environment. HIF activates genes that control oxygen homeostasis in cells, including those involved in glucose metabolism and lactic acid metabolism. These molecular changes lead to glycolysis rather than oxidative metabolism to complete tumor adaptation [164].

Accurate m6A level is crucial for behavior and electrophysiological properties of mouse cortex in response to acute stress [135]. Moreover, m6A also plays a vital role in cellular response to external stimuli such as viral infection [136, 137], DNA damage [138] and heat shock response [139, 140]. For example, m6A modifications on transcripts rapidly recruit DNA polymerase to ultraviolet (UV) induced damage sites to facilitate DNA repair and cell survival [141]. m6A pathway may be important for hypoxic regulation by HIFs. Previous studies reported that hypoxic induction of ALKBH5 was dependent on HIFs and contributed to the breast cancer stem cell phenotype [142]. In the hypoxia/reoxygenation-treated cardiomyocytes, METTL3 is responsible for inhibiting autophagic flux and promoting apoptosis [143]. However, detailed regulatory mechanisms of cellular response to hypoxia by m6A pathway are still unclear.

Transcriptional activation by HIFs is the main pathway for hypoxic adaptation. Consistent with a prior report [144], a study by Wang et al. [145] observed up-regulation of ALKBH5 upon hypoxic stress. Moreover, the total m6A level in poly(A)+ RNAs was downregulated during this process. Down-regulation of total m6A levels of mRNA and protein levels of m6A readers under hypoxic condition might promote mRNA stability, reducing the need to produce new mRNAs. Upon hypoxic stress, HIF1A is stabilized, enters the nucleus, heterodimers with HIF1B to bind to the HRE elements in the promoters and activates target genes including ALKBH5. Considering that the repertoire of m6A regulator is far from complete, new m6A regulators are continually to be identified [146], we speculate that additional regulators are also involved in regulation of hypoxia. Reprogramming of m6A epitranscriptome further reshapes transcriptome and proteome to promote glycolysis and gluconeogenesis, and inhibit mitochondria oxidative respiratory chain, facilitating cells to response efficiently to hypoxia.

Moreover, many studies have reported that m6A genes participate in the formation of a hypoxic microenvironment. For example, FTO, an m6A regulator, promoted the progression of hypoxic TME formation via effects on glucose metabolism through FOXO1 mRNA expression. In turn, tumor hypoxia regulated the function of m6A reader YTHDF1 to drive the malignancy of hepatocellular carcinoma. Hypoxia has been reported to reprogram the chromatin by inducing changes in histone methylation to determine transcriptional activity, a process independent of HIFs [147, 148]. Meanwhile, m6A deposition was reported to occur cotranscriptionally guided by H3K36me3 (histone H3 trimethylation at lysine 36) [149]. Further studies of hypoxic stress are warranted to investigate whether m6A modification is involved in histone methylation-regulated transcripts, which may enhance our understandings of the molecular mechanism of hypoxia.

### Availability of data and materials

Data sharing is not applicable to this article as no datasets were generated or analysed during the current study.

## CONCLUSIONS AND PERSPECTIVES

In recent years, with in-depth research on the mechanism of tumor development and anti-tumor therapy, m6A-related ncRNAs have received increasing attention because of their key roles in tumor growth, invasion, angiogenesis, metastasis, and immune tolerance, which is mediated by TME. The interaction between ncRNA and m6A has enriched our understanding of the complex regulatory network in TME, providing a new direction for studies on the role of post-transcriptional regulation in tumors. This interaction is a potential target for tumor diagnosis, prognosis evaluation, and treatment. Currently, the understanding of the role of m6A-related ncRNAs in TME is very limited, and more studies are needed to further reveal its regulatory mechanism and biological effects. In addition, the following limitations still exist: (1) Although m6A modification is widely present in all types of RNAs, current studies mainly focus on mRNA m6A modification, and many m6A-related ncRNAs have not been identified. (2) Because the role of m6A-related ncRNAs in tumors is still an emerging field, the genetic/epigenetic heterogeneity of different tumor cell lines and samples may lead to different results, which needs to be clarified by conducting follow-up studies. m6A-related ncRNAs show different functions in different tumors, which may be related to different downstream pathways, requiring further verification. (3) There are other kinds of RNA modifications besides m6A, such as m5C(5-methylcytidine), m6Am (N6-2′-O-dimethyladenosine), Ψ (pseudouridine), m1A (1-methyladenosine), m7G (7-methylguanosine), and hm5C (5-hydroxymethylcytidine). However, only a few of these have been studied; hence, it is necessary to consider the interaction between m6A and other modifications. Changes in specific phenotypes may not be entirely caused by alterations to a single type of RNA modification. (4) In terms of clinical applications, there is still a lack of specific methods for clinical diagnosis and treatment. As potential tumor markers, the diagnostic sensitivity and specificity of m6A-related ncRNAs need to be further validated, and their reference ranges in body fluids need to be determined. Sample handling and detection methods must be standardized. As a potential anti-tumor therapeutic target, more studies are needed to evaluate its efficacy and side effects and determine the appropriate administration method and dose. The role of m6A-related ncRNAs in TME is an exciting area of research. Clarifying the molecular mechanism of interaction between m6A-related ncRNA and the microenvironment, designing targeted drugs based on its complex and fine regulatory network, and combining it with traditional anti-tumor therapies to achieve improved efficacy and minimized side effects for individual precision medicine will be a major breakthrough in cancer research.
